# Efficiency of Amino Acid Utilization in Nellore Cattle Grazing Low-Quality Forage Supplemented with Different Sources of Nitrogen

**DOI:** 10.3390/life13081622

**Published:** 2023-07-25

**Authors:** Ana Veronica Lino Dias, Juliana Duarte Messana, Yury Tatiana Granja-Salcedo, Yeison Fabian Murilo Alfonso, Lorrayny Galoro Silva, Karine Dalla Vecchia Camargo, Kênia Larissa Gomes Carvalho Alves, Paloma Helena Gonçalves, Ricardo Andrade Reis, Telma Teresinha Berchielli

**Affiliations:** 1 Department of Agricultural, Livestock and Environmental Biotechnology, School of Agricultural and Veterinary Sciences, São Paulo State University (UNESP), Jaboticabal 14884-900, SP, Brazil; anaveronica.lino@hotmail.com; 2 Department of Animal Science, School of Agricultural and Veterinary Sciences, São Paulo State University (UNESP), Jaboticabal 14884-900, SP, Brazil; ygranja@agrosavia.co (Y.T.G.-S.); yeison.murillo29@hotmail.es (Y.F.M.A.); lorraynyg@hotmail.com (L.G.S.); karinedallavecchia@hotmail.com (K.D.V.C.); kenialgcalves@hotmail.com (K.L.G.C.A.); loma.helena@hotmail.com (P.H.G.); rareis@fcav.unesp.br (R.A.R.); telma.berchielli@unesp.br (T.T.B.); 3 Corporación Colombiana de Investigación Agropecuaria, AGROSAVIA, Centro de Investigación El Nus, San Roque 053037, Antioquia, Colombia; 4 Instituto Nacional de Ciência e Tecnologia/Ciência Animal, Viçosa 36570-000, MG, Brazil

**Keywords:** corn gluten meal, digestibility, duodenal flow, Nellore, rumen undegradable protein, urea

## Abstract

This study aimed to evaluate the effects of supplementation with non-protein nitrogen (NPN) or ruminal undegradable protein (RUP) on intake, digestibility, and amino acid (AA) use efficiency of Nellore cattle grazing during the dry season. Eight Nellore steers (12 ± 2 months old) were used in quadruplicate Latin squares (2 × 2). The animals were placed on *Urochloa brizantha* cv. Xaraés under continuous grazing. The treatments included the following: (1) urea supplementation (NPN) and (2) supplementation of corn gluten meal 60 (CGM, RUP). Animals supplemented with CGM showed higher intakes of dry matter (DM) supplement, total AA, essential AA, and individual AA. The supplementation did not affect the total AA digestibility, total AA flux, and the AA fluxes of microbial origin and RUP from the diet (*p* > 0.05). The ruminal microorganism origin flux of total AA to the duodenum was 44.5% and 52.7% for animals supplemented with NPN and CGM, respectively. Animals supplemented with CGM showed an increase in blood concentrations of isoleucine (+19.09 μmol/L), cystine (+27.29 μmol/L), and albumin (+0.11 g/dL) (*p* < 0.05), but this increase was not accompanied by an improvement in N use efficiency of steers (*p* > 0.05). RUP supplementation via CGM can be an efficient nutritional strategy to enhance the intake and absorption of AA by Nellore cattle grazing low-quality forage during the dry season.

## 1. Introduction

The livestock industry worldwide is facing challenges in reducing its environmental impact [1]. Improving the efficiency of nitrogen utilization (ENU) and reducing N excretion of ruminants can contribute to decreasing nutrient excretion into the environment [2] and climate change. Several aspects of ruminant nutrition may be directly related to the inefficient utilization of dietary N by the animal, including a nutritional imbalance in the basal diet [3].

Pastures represent the basal diet of cattle in tropical countries and, therefore, play an important role in livestock production systems [4]. However, in the dry season, tropical forages available to animals present a high concentration of fiber and lignin, while crude protein (CP) content is below 7%, which is a limiting factor for microbial growth and microbial protein synthesis [5,6]. Additionally, a high fraction of the total N in the plant may be associated with the cell wall, which may result in low rumen ammonia nitrogen (RAN) synthesis [7] and, consequently, low microbial protein synthesis.

The N imbalance in tropical forages can be corrected by supplementing animals with non-protein nitrogen (NPN) and rumen undegraded protein (RUP). NPN supplementation improves the efficiency of microbial protein synthesis and increases the supply of AA microbial to the small intestine (SI). However, NPN utilization in the rumen depends on the available energy [2,3,6,8]. In turn, RUP supplementation is known to increase AA flow to the SI, and its use by animals is not associated with the energy available in the rumen. Studies have demonstrated increases in N retention, N recycling, and animal performance when the dietary rumen degradable protein (RDP) concentration is low [7,9,10]. AA supply to ruminants is dependent on microbial protein synthesis in the rumen, the protein that escapes ruminal degradation (RUP) and reaches the SI, and the profile and digestibility of AA [11,12]. However, the efficiency of N utilization is dependent on the profile and digestibility of AA in the SI, which is known to be affected by diet [10,13].

Corn gluten meal (CGM) is feed known to have a relatively high concentration of RUP, approximately 60% [14]. However, it is important to note that CGM, similar to corn, has an unbalanced AA profile, with a high concentration of Met, Leu, and Pro and a low concentration of Lys [14,15]. Nevertheless, the AA provided by the RUP fraction may have a different profile from that of the original feed [15]. There is also a difference between the amount and digestibility of AA from microbial proteins and the RUP fraction, where the true intestinal digestibilities of total, essential, and nonessential AA, lysine, and methionine were 75.0%, 77.0%, 74.0%, 77.0%, and 86%, respectively, and the true intestinal digestibility of total microbial AA was 80% [16,17]. Therefore, knowing the AA that is supplied by RUP feed sources and by the microbial protein and knowing the flow and digestibility of each individual AA is of essential importance to meet the animal’s requirements [13] and to improve the efficiency of CP use [18]. In addition, such information may contribute to improving the prediction of N use efficiency, which is scarce in the literature on beef cattle grazing low-quality forage. Furthermore, the models used in ruminant nutrition, such as NRC [19], BR-Corte [20], and NASEM [21], consider that the AA profiles of feed and RUP are similar, as well as the digestibility of individual AA, which is not true [10].

Therefore, this study aimed to evaluate the effects of supplementation with NPN and RUP on the intake, digestibility, flow, and AA use of Nellore cattle grazing low-quality forage during the dry season. We hypothesized that NPN supplementation would increase microbial protein synthesis and that RUP supplementation would increase the AA flow and absorption in the small intestine.

## 2. Materials and Methods

The experimental procedures used in this experiment followed the animal care and handling by the Brazilian College of Animal Experimentation (COBEA—College of Animal Experimentation Guidelines) guidelines and was approved by the Ethics, Bioethics, and Animal Welfare Committee of the São Paulo State University (Unesp Jaboticabal, SP, Brazil, protocol #16.688/16).

This study was conducted at the Department of Animal Science of School of Agricultural and Veterinarian Sciences (FCAV) of UNESP-Jaboticabal (21°15′22″ S and 48°18′58″ W at 595 m altitude) during the dry season from September to October. According to the Köppen International System, the climate of the region is classified as tropical with rainy summers and relatively dry winters (Aw). Minimum and maximum precipitation during the experiment were 64.9 and 157.0 mm, and the maximum and minimum temperatures were 32 and 17 °C, respectively (Agro Climatological Station—Unesp-Jaboticabal). The experimental area consisted of 8 paddocks of 1.8 hectares, each composed of *Urochloa brizantha* (A. Rich.) Stapf. cv. Xaraés (5.51% of CP and 28.7 cm of height on average).

### 2.1. Animals, Experimental Design, and Treatments

Eight castrated Nellore steers, cannulated in the rumen and duodenum, averaging 263 ± 49 kg of body weight (BW) and 12 ± 2 months old, were used. Then, animals were randomly assigned in four 2 × 2 Latin square (2 treatments and 2 periods) designs.

The experiment lasted 56 days, which consisted of two experimental periods of 28 days each (13 d for diet adaptation, 7 d for stabilization of fecal excretion of external marker, and 8 d for sample collection). Animals were kept in paddocks under continuous stocking, and the canopy height was maintained at 28.7 cm. Each paddock contained automatic water trough and covered feed bunk to offer the supplements. Animals had free access to water and received the supplement daily at 0900 a.m.

Treatments were as follows: (1) supplementation of urea as source of NPN (50% of the RDP daily requirement; NPN) and (2) supplementation of corn gluten meal 60 as source of RUP (3 g/kg of BW per day; CGM). Additionally, all animals were supplemented with mineral mix that provided the following [per kg of DM]: Ca, 160 g; P, 40 g; Mg, 5 g; S, 40 g; Na, 160 g; Cu, 945 mg; Mn, 730 mg; Zn, 3500 mg; I, 70 mg; Co, 56 mg; Se, 18 mg; F [máx] 400 mg. Nutrient requirements followed the recommendation of Valadares Filho et al. [20] to meet an average daily gain (ADG) of 0.350 kg/animal with the following requirements of (kg/d): DM = 4.58 kg, TDN = 2.82 kg/DM, CP = 0.56 kg/DM, RDP = 67.86% of CP.

Supplements samples were collected every three days, and forage samples were collected every 28 days using the hand-plucking technique [22]. Forage samples were weighted and dried under forced air (55 °C for 72 h). The chemical composition of the supplement and forage is shown in Table 1.

### 2.2. Feed Intake, Digestibility, and Nitrogen Balance

Forage intake and fecal production were determined using two markers. Chromium oxide (Cr_2_O_3_) was the external marker used to determine fecal production, and for that, 8 g/animal of Cr_2_O_3_ was daily administered to the animals via a rubber tube directly placed into the esophagus of the animal. The Cr_2_O_3_ administration was performed at the time of supplementation (0900 a.m.) for 12 days, which consisted of 7 days for stabilization of fecal excretion of the marker and 5 d for sample collection. Fecal samples were collected at 0900, 1300, 1700, 2100, and 0600 h, on the first, second, third, fourth, and fifth day of sample collection, respectively. The samples were dried in forced air (72 h at 55 °C); half of the sample was ground (Wiley mill; Thomas Scientific, Swedesboro, NJ, USA) to pass a 1 mm sieve, and the other half to pass a 2 mm sieve.

Forage DM intake (DMI) was estimated based on fecal production and using the indigestible neutral detergent fiber (iNDF) as the internal maker. Samples of feces, supplement, and forage (from manual simulation of grazing) were dried (55 °C for 72 h) under forced air and ground to pass through a 2 mm screen sieve in a Wiley mill (Thomas Scientific, Swedesboro, NJ, USA). The samples were then weighted, placed into ANKOM bags (Filter bag F57; ANKOM Technology Corporation, Fairport, NY, USA), and incubated in cannulated Nellore animals for 288 h [24]. The NDF concentration of the bags was determined using Ankom200 Fiber Analyzer (Ankom Technology, Fairport, NY, USA), and DMI was calculated by the sum of forage and supplement intake. The duodenal flow of DM was estimated using iNDF as an internal marker according to Udén et al. [25].

Spot urine samples were collected from the steers via spontaneous urination, two hours before and four hours after supplementation, approximately on days 24, 26, and 28 of each experimental period, and used to determine the microbial protein yield and N balance. Samples were filtered in two-layer cheesecloth (10 mL each) and diluted with 40 mL of H_2_SO_4_ solution (0.036 N) to avoid bacterial degradation of purine derivatives and uric acid precipitation [26]. Subsequently, the samples were used to quantify urinary levels of urea, nitrogen, creatinine, and allantoin.

On day 28 of each experimental period, ruminal content was collected at 0, 6, 12, 18, and 24 h after supplementation. Samples were obtained from the dorsal, medial, and ventral regions of the rumen via the ruminal cannula and were strained through two layers of cheesecloth. Then, a 40 mL subsample was taken and stored at −20 °C for further analysis of ruminal NH_3_-N concentration.

### 2.3. Duodenal Flow

On days 27 and 28 of each experimental period, duodenal digesta samples were collected via duodenal cannula at 0200, 0800, 1400, and 2000 h on day one and 0500, 1100, 1700, and 2300 h on day two. Samples were kept at −20 °C, and at the end of the period, samples from each animal were pooled to form a composite sample per treatment per period. Soon after collection, 50% of the duodenal samples were dried (55 °C for 72 h) under forced air, ground in a Wiley mill (Thomas Scientific), and stored for further chemical analysis and iNDF determinations. The remaining 50% of the duodenal samples were frozen at −15 °C for further bacterial isolation.

Bacterial isolation from the duodenal digesta was performed following the method described by Cecava et al. [27]. The composite digesta samples were filtered through a 100 μm nylon filter (44% pore surface area; Sefar Nitex 100/44, Sefar, Thal, Switzerland), and the retained material was washed with saline solution (800 mL of 0.9% NaCl [weight/vol]). Then, the microorganisms were isolated from the filtered sample via centrifugation. Samples were centrifuged (2000 rpm for 20 min at 5 °C), and the supernatant was separated and centrifuged (12,340 rpm for 20 min at 5 °C). The supernatant was discarded, and 100 mL of 0.9% saline solution was added to the pellet. Then, samples were centrifuged (12,340 rpm for 20 min at 5 °C), and the resulting pellet was collected, frozen (−80 °C), freeze dried for 72 h, and used for AA profile analysis.

### 2.4. Blood Parameters

Blood samples from the jugular vein were collected from all animals (after 16 h of solid fast) at 0800 and 1600 h on day 28. The blood samples were collected in vacutainer tubes (10 mL; BD Biosciences, Franklin Lakes, NJ, USA) coated with heparin (143 IU), centrifuged (1000 g for 20 min at 4 °C), and the plasma was collected and stored (−20 °C) for further analysis. The total protein, albumin, and urea concentrations were determined using colorimetric method (Labmax 100, S.A., Lagoa Santa, Brazil) using commercial kits (Labtest^®^, Lagoa Santa, Brazil).

### 2.5. Analysis of Amino Acids

The AA compositions of feed, blood, feces, and microbial pellet samples were determined according to Hagen et al. [28] using high-performance liquid chromatography (HPLC) using an amino acid analyzer (SPC 1000, Waters Corporation, Milford, MA, USA) fitted with a pre-column derivatization system containing phenyl isothiocyanate (PITC) and a silica column (LUNA, C18 100 Å 5 u, 250 × 4.6 mm, Code 00G-4252-EQ) of reverse phase and UV detection (254 nm).

Hydrolysis of the samples (200 mg) was performed with a 9 mL 6N HCl solution containing 3% (wt/vol) phenol in sealed tubes under vacuum in a thermal reaction block for 24 h at 110 °C. Then, an aliquot of an α-aminobutyric acid internal standard was added. Samples were dried (70 millitorrs in a cryogenic nitrogen trap system), neutralized (4:4:2 solution of 0.2 N sodium acetate trihydrate, HPLC grade methanol, and triethylamine), and dried again as previously described. Subsequently, PITC was added to derivatize the AA released via hydrolysis and form the AA-PITC. Then, a 500 µL of mobile phase A was added as a diluent to the tube containing the derivatized AA crystals, and the ultraviolet detection was performed at 254 nm after reverse phase chromatography (30 uL injection loop, pH 6.40, binary linear gradient with flow of 1 mL/min, and column temperature of 58 °C). The mobile phase A was composed of 0.14 N sodium acetate buffer, acetonitrile (240 mL/2000 mL of 0.14 N sodium acetate), and triethylamine (1 mL/2000 mL of 0.14 N sodium acetate). The mobile phase B was composed of a 6:4 solution of acetonitrile (HPLC grade) and milli-Q water.

Plasma AA concentration analysis was performed by mixing plasma (200 μL) with a 0.1 N HCl solution (50 μL) containing alpha-aminobutyric acid as an internal standard and methanol 99% (250 μL). The samples were homogenized by vortexing for 10 s, centrifuged (13,000× g for 10 min at 4 °C), and dried (vacuum station to 70 millitorrs). Then, 20 μL of PITC-containing derivatization solution was added, and the samples were vortexed for 10 s, left to rest for 20 min, and dried again. Subsequently, 500 μL of diluent were added to the derivatized AA crystals, and the samples were then kept for 10 min under ultrasound, homogenized by vortexing for 15 s, and filtered through 0.45 μm Millex into a flask.

### 2.6. Chemical Analysis and Calculations

Feed samples were analyzed for concentrations of DM (method 934.01), OM (method 942.05), and ether extract (EE; method 954.02) according to AOAC [29]. The NDF concentration was determined according to Mertens [30] and adapted to Ankom200 Fiber Analyzer, corrected for ash and protein later. The CP concentration was determined using the Kjeldahl procedure according to AOAC [29].

Urine samples were used for determination of purine derivatives concentration using colorimetric method [31], total N via the Kjeldahl method [29], creatinine and urea via Labtest biochemical analyzer (Labmax 100, S.A., Lagoa Santa, Brazil), and efficiency of microbial protein synthesis using method described by Zinn and Owens [32]. The Cr_2_O_3_ concentration in fecal samples was determined according to the method INCT-CA M-007/1 [33]. Ruminal NH_3_-N concentration was determined according to the methodology adopted by Fenner [34]. In brief, ruminal fluid NH_3_ was analyzed by distilling it with 2 M potassium hydroxide (KOH) in a micro-Kjeldahl.

The total duodenal flow (g/d) of each individual AA was calculated by multiplying the respective concentration (g/kg) in the digesta sample by the DM flow. Flows of microbial AA (g/d) to the duodenum were estimated based on the respective microbial protein flow and AA composition of bacterial samples isolated from ruminal contents. The duodenal flow (g/d) of each individual AA from RUP origin was calculated by subtracting the duodenal flow of each microbial AA from the total flow of each AA. The digestibility of each individual AA (g/kg of DM) was calculated by subtracting the AA excreted in feces from the total duodenal flow of AA, dividing this by the total duodenal flow of AA multiplied by 1000.

### 2.7. Statistical Analysis

All data were subjected to least squares ANOVA using the R software version 3.6.3 (R Core Team, 2015) after verification of normality (Shapiro–Wilk test) and homoscedasticity (Bartlett test). The intake, apparent digestibility, and duodenal flow of AA, and nitrogen balance data were in a quadruplicate 2 × 2 Latin square design. The model considered treatment as fixed effects and Latin square, animal, period, and residues as random effects. The NH_3_-N concentration and blood parameters data were analyzed as repeated measures in a quadruplicate 2 × 2 Latin square design. The model included the fixed effects of treatment, time, the interaction between treatment and time, and the random effects of Latin square, animal, period, and residues. Tukey test was conducted to assess statistical significance (*p* ≤ 0.05) and tendency (0.05 < *p* ≤ 0.10).

## 3. Results

### 3.1. Intake and Duodenal Flow of Amino Acid

Treatments did not affect the intake of DM, DM forage, and CP. However, an increase in the intake of DM supplement, total AA (*p* = 0.02), EAA (*p* = 0.02), and all individual AA analyzed was observed when Nellore cattle grazing low-quality forage in the dry season were supplemented with CGM (Table 2).

Duodenal flow (g/d) of total AA, EAA, and NEAA did not differ among treatments (*p* > 0.05; Table 3). However, although the flow of total AA from ruminal microorganism origin was not affected by supplementation, its contribution to the total AA flowing to the duodenum was 44.5% and 52.7% for animals supplemented with NPN and CGM, respectively.

### 3.2. Apparent Digestibility and Nitrogen Balance

There was no interaction between time and supplementation for ruminal N-NH_3_ concentration (*p* > 0.05). The supplementation with different sources of nitrogen did not have an effect on ruminal NH_3_-N concentration, microbial-N, bacterial efficiency (kg of rumen-degraded organic matter), urinary-N excretion, and retained-N (*p* > 0.05; Table 4). However, fecal-N excretion tended (*p* = 0.06) to be greater in animals supplemented with NPN than in CGM. Supplementation with NPN or CGM did not affect the digestibility of total AA, EAA, NEAA, and the concentration of most individual AA in the SI (Table 4). Similarly, the intestinal digestibility of AA from dietary RUP (NPN) plus endogenous origin (total AA, EAA, NEAA, and the individual AA) did not differ between treatments (*p* > 0.05; Table 5).

### 3.3. Blood Parameters

There was no interaction between time and supplementation for the blood concentration of total protein, albumin, and urea (*p* > 0.05). However, serum albumin concentration was decreased (*p* < 0.05) in animals supplemented with NPN compared to CGM (Table 6). Blood urea concentration was lower at 8 h than at 20 h, with averages of 43 and 51.5 mg/dL, respectively (*p* < 0.05).

Blood concentration of the AA isoleucine and cystine was increased (*p* < 0.05) when the animals were fed CGM (Table 6). Additionally, the blood concentration of the AA threonine, alanine, and glycine tended (*p* > 0.10) to be greater in animals supplemented with CGM than in NPN animals.

## 4. Discussion

In this present study, supplementing grazing beef cattle with NPN or CGM during the dry season did not affect microbial protein synthesis or the flow of total AA to the duodenum. Therefore, the N use efficiency of the animals was similar across the treatments. The amount of each AA available to the animal varies according to the DM intake, RUP composition, microbial protein, and intestinal digestibility of each individual AA [35,36]. In addition, differences in ruminant intermediary metabolism and feed transformation due to ruminal fermentation also interfere with the amount of AA available for absorption by the animal [37].

The CP intake was similar between the two supplementation strategies tested in this study. However, there were variations in the intake of individual AA, total AA, EAA, and NEAA across treatments. These differences were primarily due to variations in the intake of forage and supplements, where the supplements containing CGM resulted in increased DM supplement intake and total AA intake. It is important to note that measuring AA intake alone may not accurately reflect the actual intestinal supply of AA in ruminants due to the transformations that occur in the rumen. However, the variation in the AA profile of the consumed CP plays a significant role in the contribution of N supply to microbial protein synthesis in the rumen.

In addition to the DMI, the amount of OM degraded in the rumen also affects the impact of dietary NNP or RUP supplementation on the flow of AA to the duodenum. Previous studies have demonstrated that the most effective mechanism for altering the intestinal availability of AA to cattle is by modifying DMI since it affects both the synthesis of microbial protein and the amount of dietary CP that escapes from ruminal degradation [11,38,39]. A deficiency of energy and protein in diets can decrease microbial protein synthesis in the rumen and the passage of AA to the small intestine. Thus, the intake of diets with protein and carbohydrates that are not degraded in the rumen increases the amount of dietary CP that passes into the small intestine but may decrease the amount of microbial protein that is synthesized in the rumen. Furthermore, Detmann et al. [40] reported that nitrogen supplementation in animals fed low-quality forage increased the intake and ruminal degradation of fiber, thereby promoting the growth of fibrolytic bacteria.

In this present study, even though the intake of total AA, EAA, and NEAA was greater in cattle-fed CGM, the digestibility and duodenal flow of total AA, EAA, and NEAA were similar between supplementation strategies. It is important to note that the animals in this study were fed tropical forage with high productive potential. However, during the dry period of the year, this plant has low efficiency of utilization by animals. This is due to the increased lignification of the cell wall during this season, which reduces forage intake and digestibility and, consequently, limits available energy to microbial protein synthesis and AA flow to the duodenum. Additionally, unlike other cultivars of the same genus, *Urochloa brizantha* cv Xaraés has additional lignification sites that affect the nutritional quality of the forage [41].

In cases of low intake of CP, microbial protein synthesis can be limited due to the scarcity of RDP available for microbial growth. This limitation subsequently leads to reduced rumen fermentation and DM intake [12,42]. However, the concentration of NH_3_-N in the rumen, which was around 23.7 mg/dL in both supplement diets, indicates optimal NH_3_-N levels for microbial growth [43]. Nonetheless, it suggests that there may be low energy available from forage to utilize the ruminal NH_3_-N. The supplementation with urea did not result in better synchronization of carbohydrate and protein digestion in the rumen.

Li [12] demonstrated that low dietary CP content and the consequent shortage of RDP limited microbial protein synthesis and resulted in a decrease in ruminal fermentation and DM intake in heifers. In this study, urea was used as a source of NPN with the objective of decreasing the possible limitations of microbial protein synthesis, which was proven by the NH_3_-N concentration in the rumen. However, the results demonstrated that urea supplementation was not effective in promoting an increase in microbial protein synthesis. One possible reason for this could be the chemical composition of the forage used, which showed an extremely low digestibility potential during the dry season, thus reducing the energy available for microbial protein synthesis [44]. Although there was no significant difference in the microbial N concentration and flow of AA from microbial origin between treatments groups, the animals supplemented with NPN showed microbial N contributing to 44.51% of the total AA flow to the duodenum, while in cattle supplemented with RUP, microbial N contributed with 52.78%. Zhao et al. [13] reported that in forage-based diets, the flow of AA to the duodenum is low due to a decrease in the flow of AA from microbial origin caused by the high lignification of the dietary fiber. This caused a decrease in the use of available N for microbial growth. The microbial N contribution observed in this study was within the range of 34–89% of the protein flowing to the duodenum, as reported by Clark et al. [11] and NRC [45].

An increase in the flow of AA from dietary RUP origin to the intestine was expected in animals supplemented with CGM. However, contrary to what was hypothesized, there was no difference in the flow of AA to the duodenum across treatments, which may be attributed to the low intake of N [7]. Similar results were observed by Cecava and Parker [46], who found no effect on the intestinal supply of AA in cattle-fed RDP and RUP.

The digestibility of AA is not constant across diets with different concentrations of CP and energy [47,48]. A high degradability of dietary CP in the rumen increases microbial synthesis [49], while a lower degradation of CP in the rumen can increase the flow of AA to the small intestine [50]. However, changes in rumen degradation are affected by the shortage of digestible energy [51]. In this study, the digestibility of AA was similar between treatments because the supply of AA that reached the intestine for digestion was similar. This was due to the microbial protein synthesis in the rumen resulting from NPN supplementation and the presence of AA from the RUP.

The results of this study differ from those observed by Souza et al. [10], who found that feeding cattle with different protein sources resulted in a distinct AA profile in microbial protein. NPN, being readily available in the rumen, tends to increase microbial protein synthesis, leading to higher concentrations of AA from microbial origin that reach the intestine. On the other hand, RUP provides AA that is not degraded in the rumen and flows to the intestine without undergoing modification in the rumen. However, in this study, the supplementation of NPN via urea was not effective in increasing the supply of AA from microbial origin to the duodenum compared to the RUP supplementation. This could be attributed to the fact that microbial synthesis is dependent on the CP intake and energy availability in the rumen.

Interpreting the results of blood AA composition can be challenging, as noted by Batista et al. [7]. Richardson and Hatfield [52] identified methionine, lysine, and threonine as the first three limiting AA used for growing cattle. According to Gibb et al. [53], a decrease in the concentration of certain AA in the blood would indicate that they are limiting. Although there were no differences in the total flow of AA to the duodenum between the supplementation groups, animals supplemented with CGM showed higher concentrations of isoleucine, cystine, threonine, alanine, and glycine in their blood. This suggests positive effects on the absorption of this AA compared to NPN supplementation. Conversely, the reduction in these AA in the plasma of animals receiving NPN indicates a greater utilization of these AA, possibly due to their limitation in protein synthesis in body tissues [53].

In this study, animals supplemented with CGM showed higher albumin levels, possibly due to the specific AA profile of this supplement. The albumin concentration is considered a long-term indicator of dietary protein content, acting as a protein reserve and AA transporter [54]. Blood urea concentration is an important indicator of protein metabolism, as it is synthesized in proportion to the NH_3_ produced in the rumen and is influenced by dietary protein and protein, i.e., energy ratio [55]. It is worth noting that blood urea concentrations between 13 and 15 mg/dL indicate a threshold for protein loss [56]. In the cases where energy-deficient animals display high blood urea levels, it may suggest a negative energy balance, leading to the utilization of AA for energy [57]. Additionally, it may imply a deficiency in AA, as certain AAs may limit the utilization of others. These factors could explain the observed results in the present study, where high blood urea concentrations were accompanied by low retention of N, indicating the catabolism of AA to meet the energy requirement of the animals, resulting in the NH_3_ accumulation [58].

The low N retention values observed in this study confirmed the dependence of energy availability on metabolism to improve N retention. Schroeder and Titgemeyer [59], in a review investigating the interaction between protein and energy supply on protein utilization in cattle, reported that although the results were variable, most of the studies demonstrated that the efficiency of protein utilization is affected by dietary energy supply. Therefore, in this study, as well as in the study conducted by Lazzarini [60], it was demonstrated that N supplementation did not increase N retention in the body of the animals, regardless of the N source used. In addition, Atkinson et al. [61], while evaluating the effects of supplemental RDP and RUP on diet digestion in lambs fed low-quality forage, reported that the ruminal degradability of protein in the diet has minimal effects on intake or ruminal fermentation. However, the authors suggested the benefit of increasing dietary RUP to improve total tract digestion of nutrients by facilitating the recycling of endogenous N. These results could be attributed to the site of digestion, as the main part of digestion and absorption of ruminants occurs in the abomasum and small intestines. The authors also recommended that ruminal N status could be improved by incorporating a component of ruminal protein degradability into prediction models. However, the results regarding AA flow and digestibility can be utilized to develop future feed formulation models that consider the variation between different diets and specific coefficient factors for AA digestibility and the efficiency of individual utilization under specific conditions [10].

## 5. Conclusions

The results of this study indicate that the hypothesized increase in microbial protein synthesis with NPN supplementation and the increase in total AA flow to the duodenum with CGM supplementation were not observed in low-quality forage diets base. However, CGM supplementation did lead to increased absorption of specific amino acids, including isoleucine, threonine, alanine, cystine, and glycine, in the cattle. This suggests that RUP supplementation via CGM can be an efficient nutritional strategy to enhance the intake and absorption of AA by Nellore cattle grazing low-quality forage during the dry season.

## Figures and Tables

**Table 1:** Chemical composition of the experimental forage and supplements.

		Supplement	
Item	Forage ^1^	NPN	CGM
Chemical composition ^2^, % of DM			
DM, %	75.6	93.9	91.2
OM	94.1	99.7	97.4
NDF	70.4	-	7.98
ADF	37.1	-	2.22
iNDF	30.3	-	1.71
NFC	16.8	-	28.9
EE	1.29	-	1.71
CP	5.51	275	58.7
RDP ^3^, % of CP	63.3	100	30.3
RUP ^3^, % of CP	36.7	-	69.7
Essential amino acids (EAA), % of DM			
Arginine	0.16	-	2.17
Histidine	0.06	-	1.49
Isoleucine	0.15	-	2.82
Leucine	0.29	-	10.9
Lysine	0.19	-	1.41
Methionine	0.02	-	1.32
Phenylalanine	0.16	-	3.98
Threonine	0.18	-	2.40
Valine	0.21	-	3.19
Non-essential AA (NEAA), % of DM			
Alanine	0.27	-	6.03
Aspartic Acid	0.39	-	5.33
Cystine	0.03	-	1.14
Glutamic Acid	0.47	-	15.7
Glycine	0.23	-	1.95
Proline	0.28	-	6.21
Serine	0.21	-	3.95
Tyrosine	0.08	-	3.37

^1^ Forage *Urochloa brizantha* cv. Xaraés; NPN = Supplementation of urea as a source of NPN (50% of the daily requirement of RDP); CGM = supplementation of corn gluten meal 60 as the source of RUP (3 g/kg of BW per day). ^2^ DM = dry matter; OM = organic matter; NDF = neutral detergent fiber assayed with a heat stable amylase and expressed exclusive of residual ash; ADF = acid detergent fiber; iNDF = indigestible NDF; NFC = non-fiber carbohydrates; EE = ether extract; CP = crude protein; RDP = rumen degradable protein; and RUP = rumen undegradable protein; ^3^ RDP and RUP content were estimated based on the protein fractions [23] and the degradation rate of each fraction, considering a passage rate of 3% h^−1^.

**Table 2:** Effects of supplementation with different sources of nitrogen on the intake of Nellore cattle grazing low-quality forage in the dry season (g/d, unless otherwise stated).

	Supplement ^1^		SEM ^2^	*p*-Value
Item ^3^	NPN	CGM		
n	8	8		
DM, kg/d	2.75	2.61	0.182	0.910
DM supplement, kg/d	0.10	0.34	0.043	0.013
DM forage, kg/d	2.65	2.26	0.179	0.244
CP, kg/d	0.43	0.34	0.034	0.516
Essential amino acids (EAA)				
Arginine	5.95	12.1	1.749	0.019
Histidine	2.73	7.27	1.161	0.015
Isoleucine	6.14	14.4	2.291	0.019
Leucine	14.3	48.8	8.466	0.015
Lysine	6.40	9.90	1.287	0.035
Methionine	1.27	5.54	1.050	0.016
Phenylalanine	6.95	19.2	3.127	0.016
Threonine	6.96	13.6	1.991	0.023
Valine	7.95	17.2	2.614	0.020
Non-essential AA (NEAA)				
Alanine	11.2	29.5	4.813	0.017
Aspartic Acid	14.7	29.3	4.555	0.028
Cystine	1.44	5.11	0.855	0.013
Glutamic Acid	22.8	71.7	12.33	0.016
Glycine	7.90	12.9	1.749	0.032
Proline	11.3	30.5	4.654	0.013
Serine	8.63	20.2	3.180	0.019
Tyrosine	4.34	14.9	2.589	0.014
AA total	141	362	58.33	0.017
EAA	58.7	148	23.68	0.018
NEAA	82.4	214	34.64	0.017

^1^ NPN = Supplementation of urea as a source of NPN (50% of the daily requirement of RDP); CGM = supplementation of corn gluten meal 60 as the source of RUP (3 g/kg of BW per day). ^2^ SEM = standard error of the mean. ^3^ DM = dry matter; CP = crude protein.

**Table 3:** Effects of supplementation with different sources of nitrogen on the duodenal flow of amino acids (AA) of Nellore cattle grazing low-quality forage in the dry season.

	Supplement ^1^		SEM ^2^	*p*-Value
Item	NPN	CGM		
n	8	8		
Essential amino acids (EAA), g/d				
Arginine	14.9	14.6	2.228	0.885
Histidine	6.67	6.88	1.093	0.871
Isoleucine	16.6	16.4	2.472	0.969
Leucine	30.6	32.6	5.113	0.815
Lysine	22.8	21.6	2.842	0.819
Methionine	7.78	7.58	0.946	0.818
Phenylalanine	18.2	18.5	2.711	0.941
Threonine	18.8	18.2	2.619	0.914
Valine	19.8	19.6	2.893	0.983
Non-essential AA (NEAA), g/d				
Alanine	26.3	26.5	3.787	0.922
Aspartic Acid	35.5	34.5	4.486	0.834
Cystine	4.78	5.12	0.799	0.754
Glutamic Acid	43.0	46.6	7.300	0.766
Glycine	21.4	20.0	2.998	0.823
Proline	16.2	17.7	2.918	0.736
Serine	17.4	17.5	2.560	0.992
Tyrosine	14.9	15.2	2.468	0.975
Taurine	1.51	2.52	0.397	0.160
AA total	337	341	49.31	0.954
EAA	156	156	22.64	0.998
NEAA	179	183	26.85	0.932
AA from ruminal microorganism, g/d				
AA total	150	180	35.94	0.777
EAA	68.0	83.0	15.99	0.782
NEAA	82.4	97.5	20.04	0.773

^1^ NPN = Supplementation of urea as a source of NPN (50% of the daily requirement of RDP); CGM = supplementation of corn gluten meal 60 as the source of RUP (3 g/kg of BW per day). ^2^ SEM = standard error of the mean.

**Table 4:** Effects of supplementation with different sources of nitrogen on the balance of nitrogen and AA digestibility of Nellore cattle grazing low-quality forage in the dry season.

	Supplement (S) ^1^		SEM ^2^	*p*-Value ^3^		
Item	NPN	CGM		Time	S	Time × S
n	8	8				
N-NH_3_, mg/dL	24.4	23.1	0.331	<0.001	0.327	0.624
Microbial-N, g/d	26.3	30.1	6.412	-	0.844	-
Bacterial efficiency ^4^	116.9	160.7	26.89	-	0.638	-
Nitrogen balance, g/d						
Urinary-N excretion	28.2	30.6	2.636	-	0.424	-
Fecal-N excretion	35.4	32.4	0.857	-	0.056	-
Retained-N	14.9	−4.33	7.590	-	0.320	-
Essential amino acids (EAA) digestibility, g/kg of DM						
Arginine	592.9	598.6	20.18	-	0.626	-
Histidine	596.9	608.0	22.82	-	0.923	-
Isoleucine	584.0	589.8	21.73	-	0.947	-
Leucine	599.9	639.8	24.78	-	0.822	-
Lysine	670.3	661.1	15.63	-	0.691	-
Methionine	700.0	695.7	21.35	-	0.322	-
Phenylalanine	582.7	612.3	21.85	-	0.978	-
Threonine	559.9	559.0	24.06	-	0.707	-
Valine	569.9	580.4	21.26	-	0.871	-
Non-essential AA (NEAA) digestibility, g/kg of DM						
Alanine	522.4	559.5	30.90	-	0.792	-
Aspartic Acid	680.8	681.4	27.80	-	0.978	-
Cystine	652.0	683.7	21.36	-	0.952	-
Glutamic Acid	592.7	616.6	28.92	-	0.915	-
Glycine	555.4	542.1	24.57	-	0.825	-
Proline	536.3	581.4	29.33	-	0.901	-
Serine	529.1	547.3	29.97	-	0.964	-
Tyrosine	648.6	666.7	19.41	-	0.869	-
AA total, g/kg of DM	598.8	615.7	23.25	-	0.979	-
EAA, g/kg of DM	602.9	616.0	20.53	-	0.863	-
NEAA, g/kg of DM	591.5	609.8	26.22	-	0.956	-

^1^ NPN = Supplementation of urea as a source of NPN (50% of the daily requirement of RDP); CGM = supplementation of corn gluten meal 60 as the source of RUP (3 g/kg of BW per day). ^2^ SEM = standard error of the mean. ^3^ Time = effects of sampling time; S = effects of supplement; and Time × S = interaction between treatment and time. ^4^ Calculated as g of bacterial N/kg of OM truly digested.

**Table 5:** Effects of supplementation with different sources of nitrogen on digestibility of AA from dietary RUP plus endogenous origin of Nellore cattle grazing low-quality forage in the dry season (g/kg of DM).

	Supplement ^1^		SEM ^2^	*p*-Value
Item	NPN	CGM		
n	8	8		
Essential amino acids (EAA)				
Arginine	454.3	342.9	458.7	0.690
Histidine	536.7	539.2	476.9	0.960
Isoleucine	479.3	422.6	469.8	0.850
Leucine	622.8	796.1	672.6	0.796
Lysine	620.1	581.4	355.2	0.885
Methionine	595.8	545.2	354.2	0.707
Phenylalanine	528.2	521.6	519.2	0.929
Threonine	520.8	461.7	525.7	0.862
Valine	513.0	482.3	503.2	0.899
Non-essential AA (NEAA)				
Alanine	500.7	571.7	580.0	0.938
Aspartic Acid	639.0	603.1	405.1	0.906
Cystine	720.9	880.0	672.8	0.839
Glutamic Acid	578.4	678.4	543.9	0.838
Glycine	535.5	425.6	569.3	0.864
Proline	708.7	305.4	679.3	0.627
Serine	580.3	601.3	680.6	0.974
Tyrosine	550.4	787.2	669.5	0.902
AA total	572.2	577.2	489.1	0.979
EAA	552.6	535.8	464.4	0.919
NEAA	581.4	603.5	520.6	0.979

^1^ NPN = Supplementation of urea as a source of NPN (50% of the daily requirement of RDP); CGM = supplementation of corn gluten meal 60 as the source of RUP (3 g/kg of BW per day). ^2^ SEM = standard error of the mean.

**Table 6:** Effects of supplementation with different sources of nitrogen on blood concentration of total protein, albumin, urea, and AA of Nellore cattle grazing low-quality forage in the dry season.

	Supplement (S) ^1^		SEM ^2^	*p*-Value ^3^		
Item	NPN	CGM		Time	S	Time × S
n	8	8				
Total protein, g/dL	7.28	7.05	0.148	0.1382	0.287	0.366
Albumin, g/dL	2.41	2.52	0.052	0.6376	0.017	0.820
Urea, mg/dL	46.2	48.3	1.978	<0.001	0.237	0.269
Essential amino acids (EAA), μmol/L						
Arginine	81.57	94.14	3.778	-	0.487	-
Histidine	61.42	76.17	5.399	-	0.592	-
Isoleucine	45.45	64.54	3.316	-	0.050	-
Leucine	57.88	66.19	5.790	-	0.894	-
Methionine	59.10	68.75	3.845	-	0.702	-
Threonine	49.96	67.48	3.283	-	0.067	-
Valine	17.91	17.63	0.137	-	0.758	-
Non-essential (NEAA), μmol/*L*						
Alanine	81.61	110.49	6.291	-	0.074	-
Aspartic Acid	39.37	45.16	2.027	-	0.466	-
Cystine	41.37	68.66	4.426	-	0.015	-
Glutamic Acid	71.45	80.63	3.279	-	0.765	-
Glycine	13.45	19.46	1.239	-	0.095	-
Proline	161.07	201.24	10.44	-	0.338	-
Serine	35.03	45.02	2.479	-	0.121	-
Tyrosine	10.45	13.23	0.763	-	0.136	-

^1^ NPN = supplementation of urea as a source of NPN (50% of the daily requirement of RDP); CGM = supplementation of corn gluten meal 60 as the source of RUP (3 g/kg of BW per day). ^2^ SEM = standard error of the mean. ^3^ Time = effects of sampling time; S = effects of supplement; and Time × S = interaction between treatment and time.

## Data Availability

Data will be made available upon request directly to the authors.
